# Application of dielectric barrier discharge for improving food shelf life and reducing spoilage

**DOI:** 10.1038/s41598-021-96887-3

**Published:** 2021-09-28

**Authors:** Subrata Roy, Bhaswati Choudhury, Judith Johnson, Alexander Schindler-Tyka

**Affiliations:** 1 Applied Physics Research Group, Department of Mechanical and Aerospace Engineering, University of Florida, Gainesville, 32611 USA; 2 SurfPlasma, Inc., Gainesville, 32601 USA; 3 Emerging Pathogens Institute, University of Florida, Gainesville, 32610 USA

**Keywords:** Biotechnology, Microbiology, Engineering, Physics

## Abstract

Dielectric Barrier Discharge (DBD) based ozone therapy is an attractive non-thermal, additive-free and environment-friendly alternative to traditional food processing technologies. Its practical application is dependent on economical ozone generation and optimum ozone dosage. This study investigates the one-time and periodic application of a compact (48 cu. cm), lightweight (55 g), low power, low temperature, DBD ozone generator for treatment of spoilage inocula prepared from combinations of spoiled green beans, grape tomatoes, lettuce and strawberries. A one-time exposure of 126–136 ppm of average ozone concentration produced by the DBD generator over 3 min and 15 min resulted in at least 1 and 4 log reduction, respectively, in microbial colonies present in the spoilage inocula. Daily exposure of 128.7 ppm average ozone concentration over 3 min under similar conditions showed that inhibition through periodic exposure can successfully inhibit the growth of both bacteria and mold species with at least 5 log reduction of microbial colonies. Visual inspection of whole fruits and vegetables with similar 3-min daily exposure showed the potential of ozone therapy to at least double the shelf-life of food products. For the daily exposures, energy required by the DBD ozone generator was calculated as 0.39 $\pm$ 0.06 kJ/day.

## Introduction

The global challenge of food safety, preservation and storage is of great concern in today’s world. One third of the total food produced worldwide for human consumption is wasted or discarded every year amounting to 1.3 billion tons of food-waste per year^1^. This has huge environmental as well as economic repercussions arising from the wastage of resources like water, energy and soil along with unwanted green gas emissions during excess food production. In addition to resource wastage, increased number of outbreaks related to food-borne diseases caused by food spoilage from microbial and fungal contamination rises the concern of food quality for public health and safety^2^. Thus, there is a need for technologies that can effectively prevent food contamination and spoilage, reducing food wastage, while maintaining quality of food products throughout the food supply chain^3^. Despite existing food preservation techniques like freezing, dehydration, antimicrobials, additives and acidification, food spoilage resulting from contamination remains a major concern in the food industry^4^. This can be attributed to ineffectiveness of current techniques, adverse effects of harsh additives and high temperature processes on food quality and toxic residuals harmful to the environment^4^. This has led the food industry to explore alternative solutions. For example, a recent review highlights the application of renewable cellulosic nanocomposites for food packaging to avoid fossil fuel plastic pollution and potential future development of biodegradable packaging to enhance the shelf life of food products^5^. Since the food spoilage is inevitable, researchers also evaluated anaerobic digestion method for converting food waste into biogas^6^. The focus of this paper is the application of non-thermal, additive-free and environment friendly food processing technologies like non-thermal plasma-based ozone treatment^7^.

Dielectric barrier discharge (DBD) is a type of non-thermal plasma formed when an AC voltage is applied across two electrodes separated by a dielectric barrier. Atmospheric DBD involves ionization of neighboring air resulting in generation of various reactive species including ozone. In the recent years, ozone has been recognized as an attractive food safety and preservation technology due to benefits of (a) rapid decontamination of a wide range of pathogens with its strong oxidizing properties, (b) no toxic residuals like hazardous halogenated compounds on treated food, (c) easy decomposition to oxygen, (d) lower energy requirements and (e) on-site generation eliminating the need for storage of hazardous chemicals^6^. Atmospheric DBD based ozone treatment has the additional advantage of using air as the source gas which eliminates the need for oxygen or nitrogen storage tanks^8^. It is important to note here that besides ozone there are light, heat, and other excited species, such as O-, OH, O, and excited nitrogen molecules produced by the DBD. However, published literature reports that ozone is the most contributing factor for microbial decontamination among other reactive species^9^. Also, it is the longest residing species compared the other active species^10^.

Ozone is a potent decontaminant proven for treatment against a broad-spectrum of pathogens including gram-positive and gram-negative bacteria, virus, fungi, protozoa and microbial spores^11,12^. Research has shown it to be more effective than conventional food disinfectants like chlorine and potassium sorbates. In 2001, ozone was approved by the USFDA (U.S. Food and Drug Administration) as an antimicrobial food additive with the Generally Regarded as Safe (GRAS) status^13^. Application of ozone treatment in preserving food products including fruits and vegetables have been reviewed by various researchers^12,14,15^. Ozone is used as a food disinfectant in both aqueous and gaseous phases. Although, some studies suggest that aqueous ozone treatment is more effective than gaseous treatment^16^, the advantage of gaseous ozone treatment lies in direct treatment of liquids and elimination of extra energy required to generate ozonated water through bubbling or diffusion^12^. Research on gaseous ozone treatment of dried foods including cereal grains, peas and beans showed 3 log reductions in *Bacillus* spp. and *Micrococcus* depending on ozone concentration, temperature, and relative humidity conditions^17^. Gaseous ozone treatment of dried figs and date fruits was reported to result in significant reduction in pathogenic microbes including bacteria, coliform, and yeast/mold counts, at 5–10 ppm exposure for 1–5 h^18,19^. Several reports on ozone treatment of food products like the ones mentioned above can be found in literature^20–23^. Further, microbial inactivation mechanism through ozone treatment has been extensively researched on indicating disruption of cell envelope and consecutive disintegration through cell lysis as the major inactivating mechanisms^12,15^. Despite existing literature on ozone food treatment, its advantages cannot be utilized without taking two factors into consideration: (a) economical and efficient ozone generation as the ozone molecule is highly unstable and expensive to generate, and (b) effective and optimum ozone dosage in order to avoid negative impacts on food quality due to excessively high ozone concentrations^4^. The major concern with adaptation of ozone treatment lies in high ozone dosages and corresponding input power required in one-time treatments for inactivation of spoilage organisms which might negatively impact food product.

This study investigates the application of a low power, low temperature, DBD based ozone therapy for food preservation by reducing spoilage and increasing shelf-life. Both one time and periodic ozone exposures are examined by controlling the time for which the DBD reactor is powered. A two-fold approach is utilized for this purpose. The first approach—direct killing, entails one time exposure of DBD generated ozone on selected bacterial strains and cocktail of food-borne pathogens. This approach identifies the one-time 15-min exposure ozone dosage required to destroy the food spoilage micro-organisms. With concerns of one-time exposure to high ozone concentrations, a more feasible approach of inhibiting growth of spoilage organisms through regular short-term exposures is examined. Thus, the second approach-inhibition, entails daily 3-min exposures of 130 ppm average ozone concentration on food spoilage organisms. The results showed that the inhibition approach can potentially double the shelf life of food products. Examination of DBD-based ozone treatment on whole fruits and vegetables was also performed through the inhibition approach under room-temperature and refrigerated conditions. Visible examination of mold and fungus growth in the whole fruits and vegetables indicated the effect of ozone treatment for food preservation. Although ozone exposure for food preservation has been studied before, this study is unique due to application of the inoculum source, low power DBD ozone generator and exposure periods. Such a study has not been performed previously to the best of our knowledge.

## Methods and materials

### DBD ozone generation and power supply

**Figure 1:**
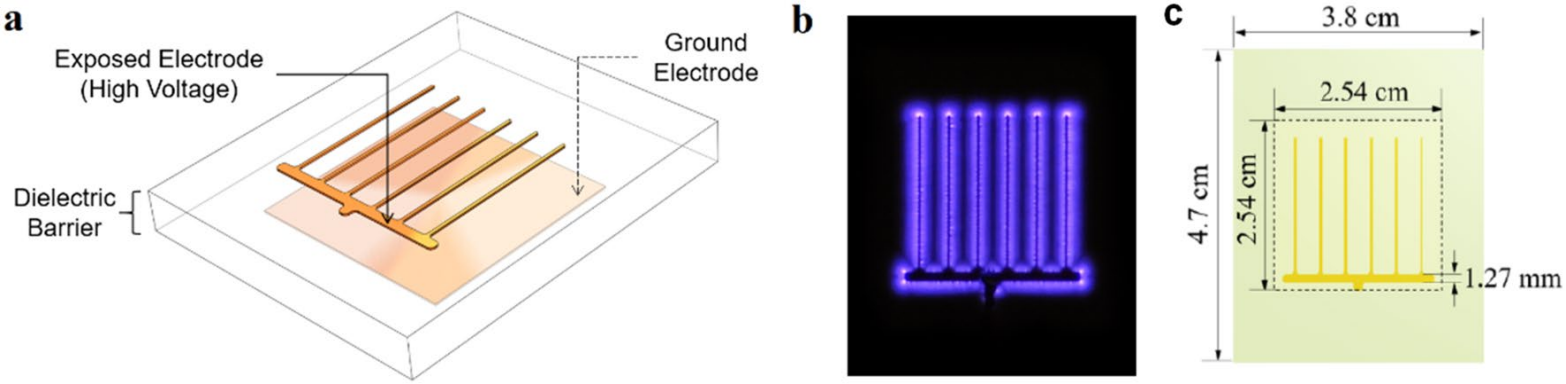
Dielectric Barrier Discharge reactor used for ozone generation (**a**) reactor showing exposed and ground electrode separated by a dielectric barrier, (**b**) plasma formed around exposed electrode when reactor is powers and (**c**) dimensions of the electrode configuration used in the reactor.

The comb electrode design shown in Fig. 1 was used to produce ozone. In this electrode configuration, discharge or plasma is formed over the surface of the dielectric barrier along the perimeter of the top electrode exposed to the surrounding air. Figure 1a shows the two copper electrodes (thickness: 35 µm) placed on the top and bottom surfaces of the dielectric material. The bottom electrode is grounded to prevent formation of plasma at the bottom electrodes. Plasma is generated around the top electrode (Fig. 1b) by applying an alternating potential difference between the top and bottom electrodes. Details of this DBD reactor can be found in previous studies published by our group on ozone water disinfection^24^.

**Figure 2:**
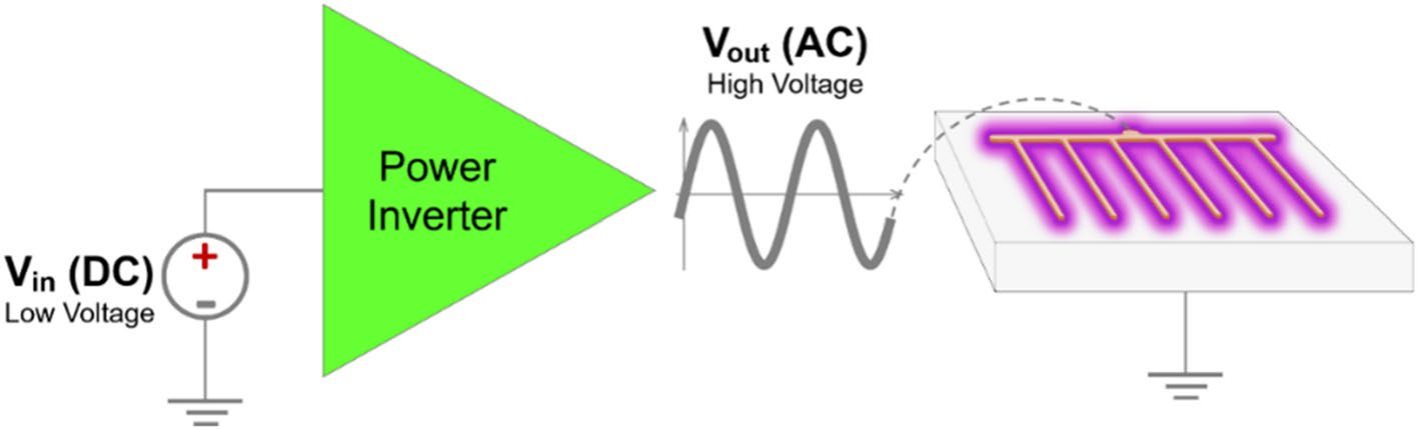
Electrical set of the Active Plasma Module to power the DBD reactor used in this study.

**Figure 3:**
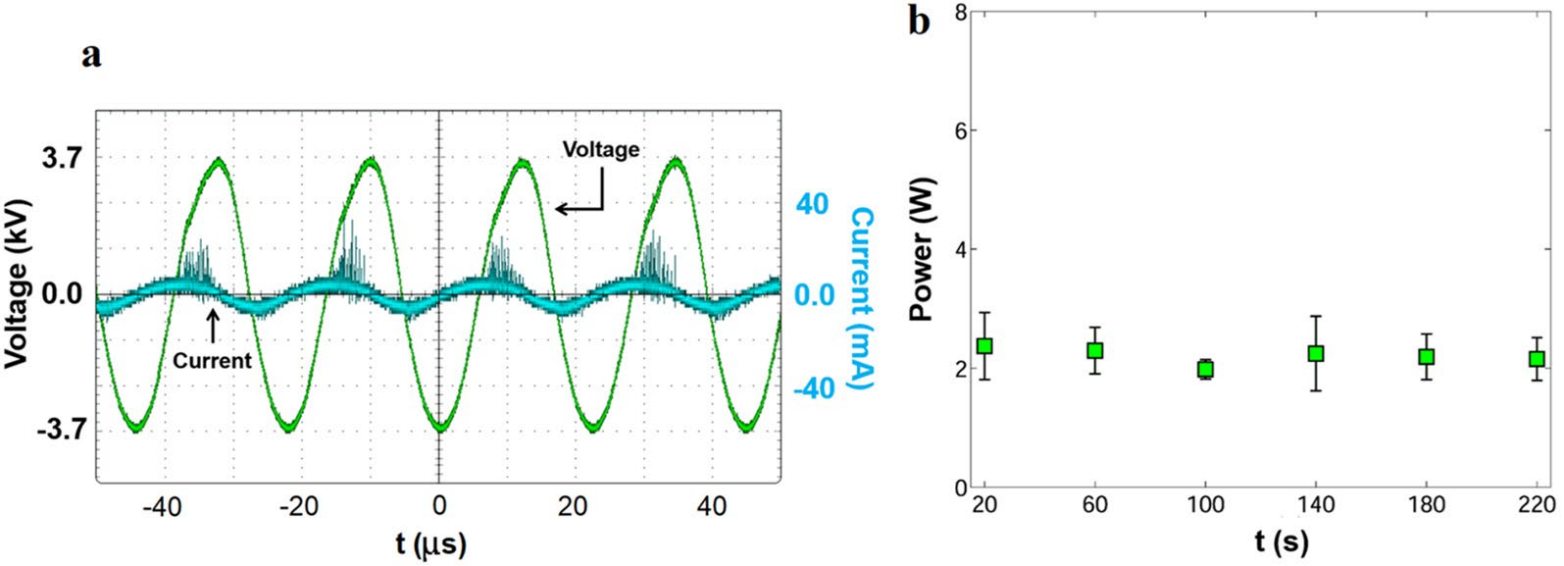
(**a**) Representative voltage and current plots, (**b**) corresponding power data.

In contrast to heavy and bulky high voltage sources usually used in DBD based ozone generation^25,26^, this study uses the Active Plasma Module (APM)^27^ which is only 48 cu. cm. in size and weighs 55 g. APM operates as a power inverter that converts a low DC input voltage into a high AC output voltage, which is then used to power the electrodes shown in Fig. 1. The electronic module was configured to work with an input voltage of 25 V (DC) proportioned by adjustable power supply model KORAD KA6005D. These input conditions generate approximately 7 kVpp (peak to peak voltage) at the output. The DBD reactor run by the APM uses a power of 2.2 ± 0.37 W^24^. A graphical explanation of this electrical system is presented in Fig. 2 and more details can be found in previously published literature by our group^24^. Representative voltage and current plots shown in Fig. 3a shows the ionization signature spikes for the reactor. We used a traditional electric current method^28,29^ to calculate power expended by the reactor as plotted in Fig. 3b.

### Preparation of cocktails of bacterial foodborne pathogens and spoilage organisms

Four different spoilage inocula were used to examine DBD based ozone therapy. One was a combination of spoiled green beans and grape tomatoes; the second green leaf lettuce and cucumber, the third was strawberries, and the fourth was a culture of *Salmonella enterica* (*S. enterica*). The first inoculum was made from 200 g of green beans and 100 g grape tomatoes. The second sample had 200 g each of butter lettuce and cucumber, and the third 200 g of strawberries. The procedure followed for preparation of these inocula was a modification of that used by Mancinelli et al.^30^, for preparing cocktails of food pathogens. The samples were placed in sterile sample bags with 200 mL of LB (Luria–Bertani) broth and 20% glycerol follwed by stomaching for 5 min. The supernatant of each sample was used as the inoculum and aliquots were frozen at − 80 °C. These produce preparations had a rich poly microbial population with both bacteria and fungi present. Recent clinical isolates were used for preparing cultures of *S. enterica*. Stocks were stored at − 80 °C in LB broth with 30% glycerol. Frozen stocks were grown overnight on LB agar plates at 37 °C followed by then dilution in LB broth for desired microbial count. To the best of our knowledge, this study comply with relevant institutional, national, and international guidelines.

### Experimental procedure

#### Direct killing approach (one time exposure)

The direct killing approach entailed one time exposure of DBD generated ozone on the four spoilage inocula prepared. This approach identifies the effect of one-time 3-min and 15-min DBD generated ozone exposure in destroying food spoilage micro-organisms. 100 µL of each prepared spoilage inocula was spread on a non-absorbent 1-inch sq. Teflon coupon. Teflon was used since it is known to be non-reactive to ozone. For each experiment, an inoculated coupon was placed in a sealed test chamber (324 cu. in.) fit with one DBD reactor for ozone generation. The inoculated coupon was then exposed to ozone for pre-determined exposure times by powering up the reactor. Another inoculated coupon was placed outside the test chamber for control count of microbial population in the inoculum. For post processing, coupons were immersed in 5 mL of L-broth and vortex to remove surviving bacteria. Serial dilutions were plated, incubated for 48 h and plated counts of exposed and un-exposed coupons were compared. Two exposure times were tested: 3 and 15 min. Three repeats were performed and averaged for each exposure time and inoculum type to have statistical confidence in the results. Control experiments were performed to verify that no ozone was lost in the chamber when Teflon coupons without any contamination were placed in the chamber. Analytical Profile Index (API) strip tests were also performed to identify the most abundant micro-organisms present in the inocula samples. The energy required to run the DBD reactor was calculated as the product of the power consumed by the reactor and the exposure time.

#### Inhibition approach (periodic exposure)

**Figure 4:**
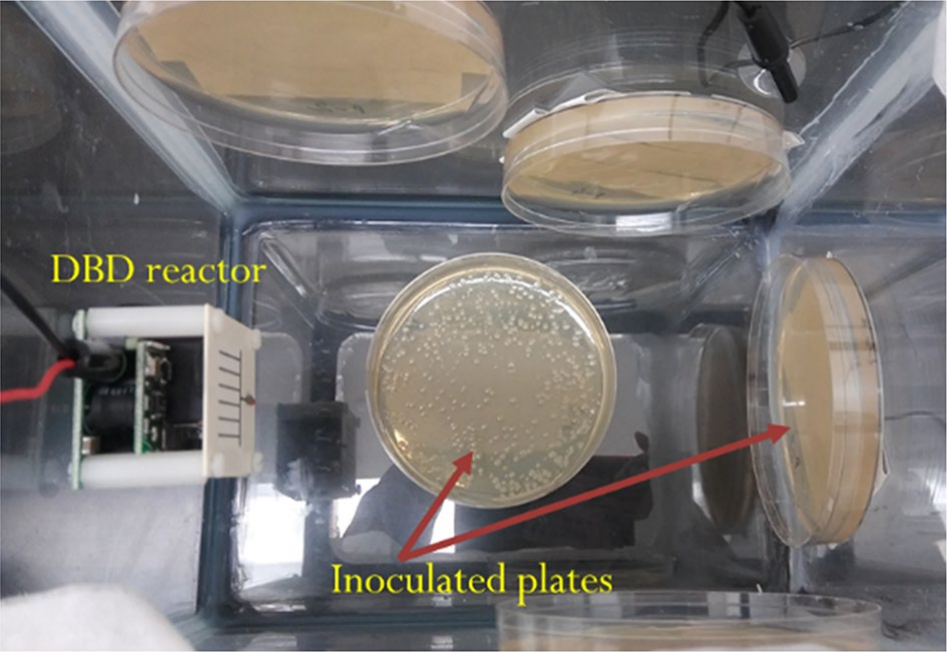
Picture of test plates inside the test chamber fitted with an APM DBD reactor for ozone generation.

The inhibition approach entailed daily 3-min ozone exposure on the four spoilage inocula prepared. This approach identifies the effect of daily 3-min DBD-generated ozone exposure in destroying food spoilage micro-organisms. For these experiments, inoculated LB agar plates were used in place of inoculated coupons used in the direct killing approach. This allowed for seeing the combined effect of inhibition of growth as well as outright killing of the organisms on bacteria growing media. Duplicate sets of appropriate serial dilutions (1:10) of the inoculum were spread on LB agar plates and one set of plates (without lids) was exposed to ozone by running the DBD reactor inside a sealed chamber as shown in Fig. 4. For the exposed test LB agar plates, the reactor was run for 3 min every day and the chamber was left closed for 24 h between ozone exposures. The plates were taken out to be photographed and counted each day before the next ozone exposure. Both sets of plates were incubated at room temperature for the duration of the experiment. The control plates that were left outside the test chamber were not exposed to ozone. The duration of these experiments was determined by the growth of mold colonies. The final day of each experiment was the day when the mold colonies were recognizable and countable. At this point, colonies were stable and not enough time had passed to allow secondary mold colonies to arise from spores. The plate count results on the final day were used to analyze the effect of periodic ozone exposure by comparing exposed and unexposed plates. Control experiments were also performed to correct for changes in LB agar plates due to ozone exposure which could affect microbial growth on those plates. The energy required to run the DBD reactor was calculated as the product of the power consumed by the reactor and the exposure time.

#### Whole fruits and vegetables (inhibition approach)

**Figure 5:**
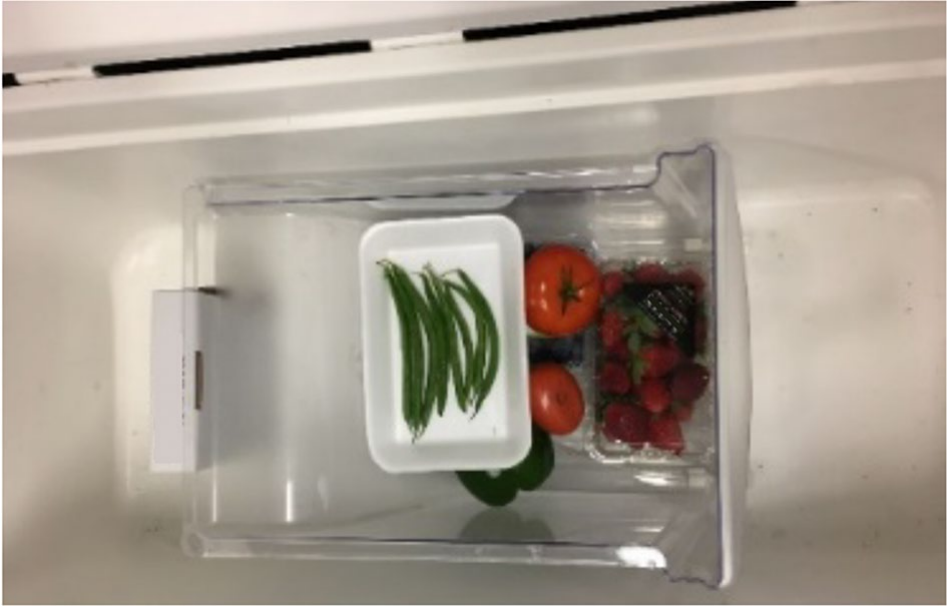
Example of selection of produce in a test chamber fit with an APM reactor.

The inhibition approach of daily 3-min ozone exposure was applied to whole fruits and vegetables to examine the practical application of this approach. For these experiments, two identical sets of produce products were placed in two containers. One of the containers was placed in a chamber fit with a DBD reactor as shown in Fig. 5. Its contents were exposed to ozone for 3 min with average concentrations of 130 ppm each day by powering the reactor. The second container was kept outside in atmospheric conditions. The produce was removed daily from both the containers to take pictures of food decomposition and then placed back in them. For examining the effect of ozone therapy under refrigerated conditions, produce was held in a standard household refrigerator with one refrigerator drawer receiving a target average amount of 100 ppm ozone by activating the DBD reactor for less than 3 min. A second identical drawer was kept in an identical refrigerator without the DBD reactor to generate ozone. A separate refrigerator was used as the drawers are not airtight and ozone generated in one drawer could affect produce in the adjacent drawer.

#### Ozone measurement

**Figure 6:**
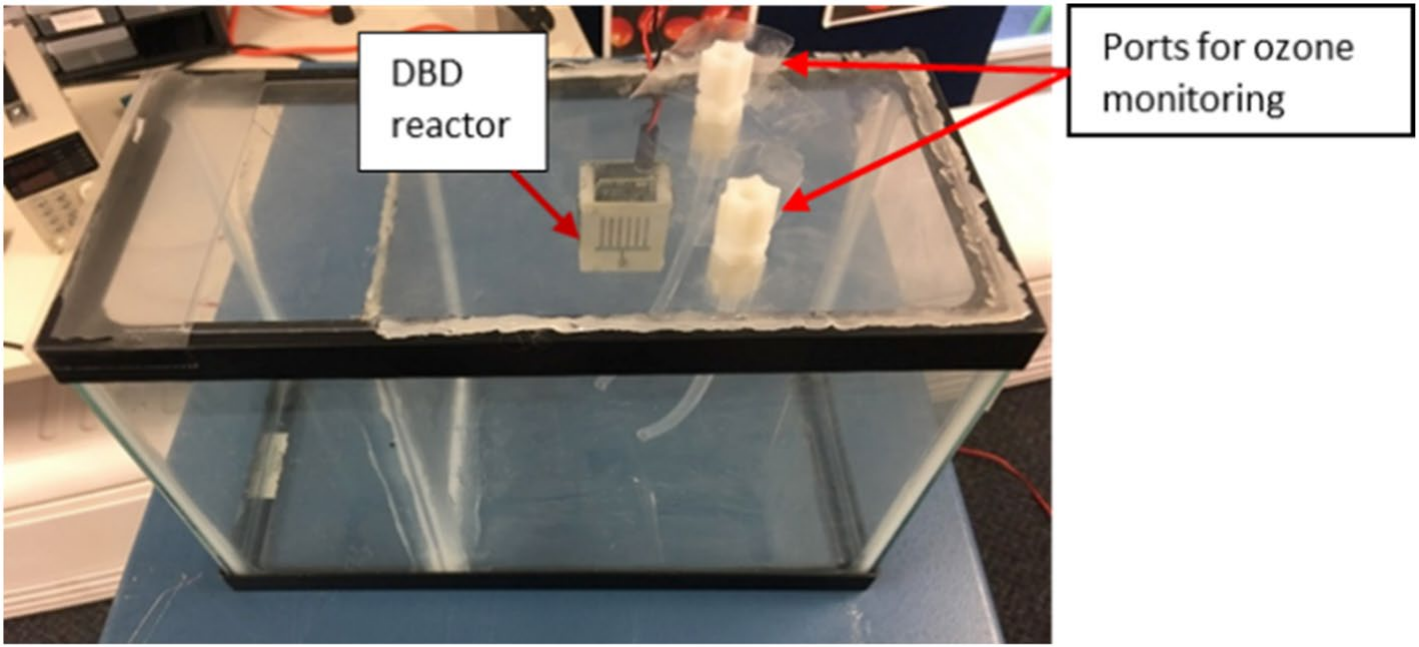
Test chamber equipped with DBD reactor and ozone probes for measuring ozone.

To determine the amount of ozone produced by the DBD reactor, it was placed in a sealed chamber identical to the treatment test chamber and connected to an ozone monitor through Teflon pipes running into the chamber as shown in Fig. 6. The experimental refrigerator was modified to allow it to be connected to the ozone analyzer as well. The 2B Technologies Model 202 Ozone Monitor™, which works based on UV light absorption at 254 nm, is used for the ozone measurements in air. The accuracy of the monitor is 1.5 ppb or 2% of the reading^31^. The ozone measurements were conducted separately without inoculated plates or coupons in the test chamber to avoid error in measurements due to ozone used up for decontamination of the inoculated plates or coupons.

## Results

In this section we present and discuss results from the DBD based ozone application to fruits and vegetables.

### Inactivation with DBD based ozone therapy

#### Direct killing approach (one-time exposure)

Averaged results from three repeats performed for determination of spoilage inactivation through one-time DBD-generated ozone exposures for 3 min and 15 min for each type of inoculum is shown in Table 1. The average control count and after exposure count of total microbial population (CFUs: colony forming units) found in the coupons was used to determine the log reduction in pathogens caused by ozone treatment of inoculum consisting of food pathogens grown from combined food spoilage.

**Table 1:** Average microbial count per coupon without (control) and with one-time ozone exposure.

Inocula name	CFUs per test coupon		
	Control	3 min	15 min
Green beans and grape tomatoes	8 × 10^5^	5.5 × 10^5^	1.7 × 10^1^
Green leaf lettuce and cucumber	1.5 × 10^5^	1.3 × 10^4^	2.9 × 10^1^
Strawberries	2.3 × 10^5^	1 × 10^4^	2 × 10^0^
*Salmonella enterica*	4.2 × 10^5^	2.1 × 10^4^	8.4 × 10^1^

**Figure 7:**
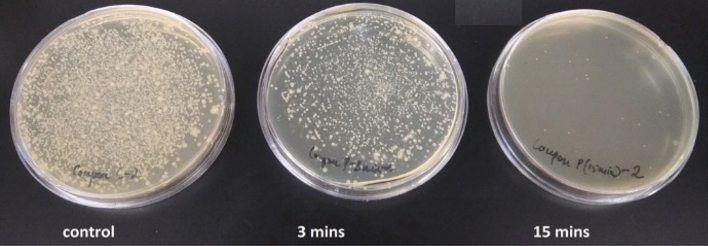
2nd dilution (1:100) plates for the control inoculum of green beans and grape tomatoes without ozone exposure and with 3- and 15-min one-time ozone exposure.

**Figure 8:**
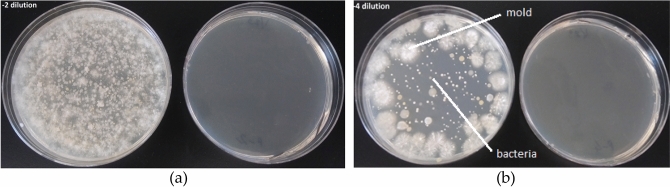
Photos of (**a**) 2nd and (**b**) 4th dilution plates for the plated inoculum of green beans and grape tomatoes without exposure (left) and 3-min daily ozone exposure (right) after 4 days.

The inocula prepared from the produce preparations had a rich poly microbial population with both bacteria and fungi as evident from the pictures of control plates in Figs. 7 and 8. With a control of 5 logs of CFUs, the 3-min exposure had limited bactericidal and fungicidal effect with a maximum of 1 log reduction in spoilage count while the 15-min exposures resulted in 4–5 log reductions.

A complete microbial work up was out of the scope of this study, but studying the different inoculum through analytical profile index or API test strips showed that the most abundant organisms in the green beans and grape tomatoes sample were *Enterobacter* sp., *Chrysobacter* sp., and an *Aspergillus* sp. The green leaf lettuce and cucumber sample had *Xanthomonas* and *Chromobacter* and the Strawberries has *Fusarium* sp. and *Enterobacter* sp. Increasing exposure time to 15 min resulted in 4–5 log reductions in total microbial population as shown in Table 1 and Fig. 7.

The averaged values of ozone measurements recorded for 3 and 15 min in the test chamber was found to be 128.7 ± 2.5 ppm and 133.5 ± 2.6 ppm. The errors were calculated based on instrument error of 2% of the reading. This implies that a one-time exposure of 126–136 ppm of average ozone concentration in a chamber of 324 cu. in. for 3 min and 15 min result in at least 1 and 4 log reduction, respectively, in food pathogens including *Enterobacter* sp., *Chrysobacter* sp., *Aspergillus* sp, *Xanthomonas, Chromobacter, Fusarium* sp. and *Enterobacter* sp. The energy required to power the reactor for 3 and 15 min was calculated to be 0.39 ± 0.06 kJ and 1.98 ± 0.33 kJ, respectively.

#### Inhibition approach (periodic exposure)

Averaged results from three repeats performed for determination of spoilage inactivation through periodic DBD-generated ozone exposures for 3 min every day (periodically over 24 h) for three types of inocula. Table 2 gives the average control count and after exposure count of total microbial population (CFUs: colony forming units) found in the inoculated plates used for examining ozone treatment of combined food spoilage.

**Table 2:** Average microbial count per inoculated plate without (control) and with periodic ozone exposure.

Inocula	CFU/plate		
	Control	Exposed	Log reduction
Green beans and grape tomatoes	3.05 × 10^8^	1.75 × 10^3^	5.28
Green leaf lettuce and cucumber	3.02 × 10^9^	2.05 × 10^2^	7
Strawberries	6.8 × 10^6^	1.04 × 10^1^	5.76

**Figure 9:**
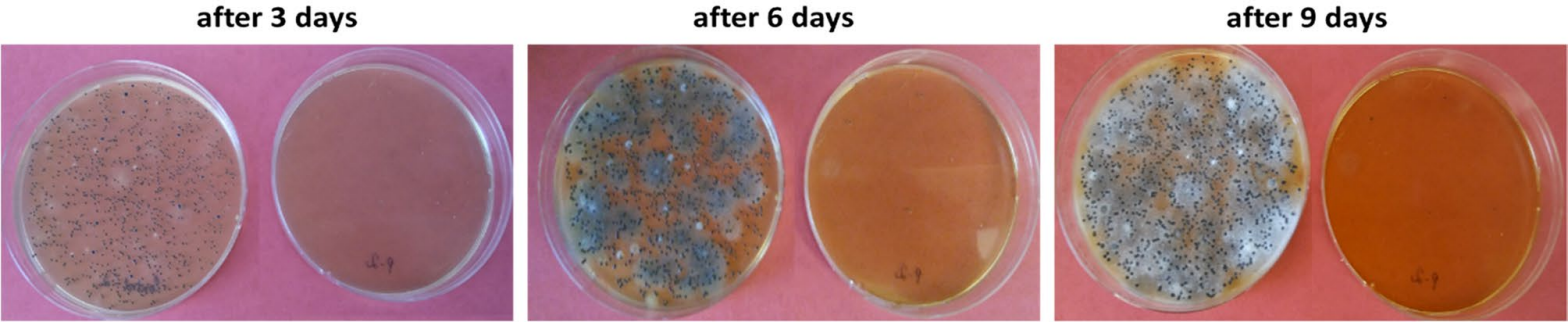
Photos of 3rd dilution plates for the plated inoculum of green beans and grape tomatoes without exposure (left) and 3-min daily ozone exposure (right) after 3, 6 and 9 days. Pink background is chosen for better visual of spores and mold.

All inocula resulted in heavy growth of flora with a mixture of microbes with spore forming mold and Gram-negative bacteria being the most common elements, see Figs. 8 and 9. The plate counts reached their maximum after 3–4 days growth, with the fungi tacking longer to appear. The 3 min per day DBD reactor generated ozone exposure through plasma activation was sufficient to produce a distinct inhibition of growth and/or killing of the organisms as evident from Table 2. The CFU of organisms exposed to ozone was always reduced, usually by more than 5 logs, after ozone exposure. We also saw a tendency for mold colonies on the ozone exposed plates to sporulate 24–48 h later than on the control plates or not sporulate at all as seen in Fig. 9. There was considerable variation in the overall reduction of colony formation. The pH of the L-agar plates remained the same after ozone exposure and the organisms grew normally on plates pre-exposed to ozone, so the inhibitory effects are not due to changes in the agar.

These ozone exposure experiments on microbes isolated from rotten green beans, tomatoes, lettuce and cucumbers, and strawberries allowed us to observe how much killing/inhibition can be achieved by exposing food spoilage inoculated plates to periodically powered DBD reactors. The experiments were performed on three types of inocula and demonstrated that periodic ozone exposure can successfully kill/inhibit the growth of both bacteria and mold species found in spoiled produce resulting in a greater than 5 log reduction of microbial colonies. Although more work is needed to determine the percentage of isolates killed versus inhibition of growth, it can be concluded that inhibition and killing of microbes is an important component of the ozone’s ability to prolong produce shelf-life with daily exposures of 128.7 ± 2.5 ppm averaged ozone concentrations over 3 min (refer “Direct killing approach (one-time exposure)”). Further, energy required to power the reactor for 3 min daily was calculated using the electric current method^29^ to be 0.39 ± 0.06 kJ/day, which is of the same order reported in the literature^9^.

### Whole fruits and vegetables shelf-life extension with inhibition approach (periodic exposure)

**Figure 10:**
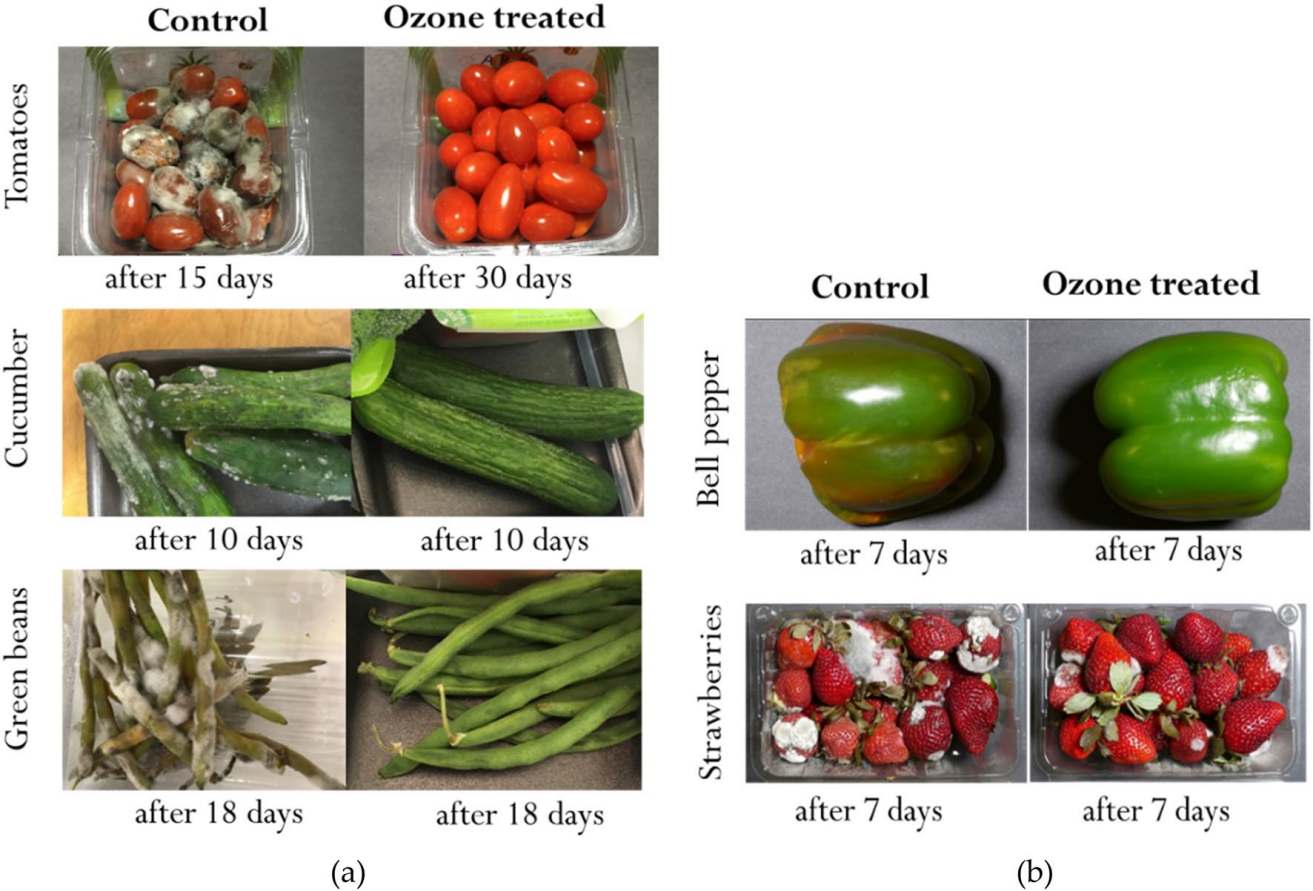
Shelf-life extension of whole fruits and vegetables at (**a**) room temperature of 21–25 °C and (**b**) under refrigeration of 5–8 °C, through DBD based ozone therapy with the inhibition approach of daily exposure of 130–150 ppm avg ozone concentration for 3 min.

The results of the experiments performed to examine the effect of the inhibition approach through daily exposure of DBD-generated ozone for 3 min on the shelf-life of whole fruits and vegetables, with and without refrigeration, is shown in Fig. 10. Visual inspection showed that the shelf life of all produce was atleast doubled in all the experiments. Considerable inhibition of mold and fungus growth in all produce was also observed in all the ozone treated produce samples in comparison to untreated samples. This can be related to the inhibition of microbial growth observed in the results of plated inoculum ozone exposure presented in “Inhibition approach (periodic exposure)” (Figs. 7 and 8). Thus, a daily exposure of 128.7 ± 2.5 ppm averaged ozone concentrations over 3 min with the DBD-reactor consuming energy of 0.39 ± 0.06 kJ/day can potentially extend produce shelf-life by more than 50%. A complete study of the effect of periodic ozone exposure on the sensor and nutritious value of produce products was out of the scope of this study. However, with previous studies showing no harmful effect of ozone exposures of 4 ppm every 3 h^32^ and one time 10-min exposure of 10 ppm^33^ ozone on food products like tomatoes shows the potential of safe and energy-efficient produce shelf-life increment through ozone therapy using the DBD reactor used in this study.

## Conclusions

This study investigated the application of a low power, low temperature, DBD based ozone therapy for food preservation using a lightweight (55 g), compact (48 cu. cm.) surface DBD reactor as the ozone generator. Both one time and periodic ozone exposures were examined by controlling the time for which the DBD reactor is powered. Exposure effects were determined on different inoculum prepared from combined and individual produce spoilage from green beans, grape tomatoes, lettuce and strawberry. Although ozone exposure for food preservation has been studied before, this study is unique due to the inoculum source, low power DBD ozone generator and exposure periods. Such a study has not been performed previously to the best of our knowledge.

A one-time exposure of 126–136 ppm of average ozone concentration in a chamber of 324 cu. in. for 3 min and 15 min resulted in at least 1 and 4 log reduction, respectively, in pathogens present in inoculum prepared from green beans, tomatoes, strawberries and lettuce spoilage. Preliminary microbial analysis showed that the most abundant micobial species in the prepared inocula were *Enterobacter* sp., *Chrysobacter* sp., *Aspergillus* sp, *Xanthomonas, Chromobacter, Fusarium* sp. and *Enterobacter* sp. The energy required to power the reactor for 3 and 15 min was calculated to be 0.39 ± 0.06 kJ and 1.98 ± 0.33 kJ, respectively.

With concerns of one-time exposure to high ozone concentrations, a more feasible approach of inhibiting growth of spoilage organisms through periodic ozone exposures was examined. This approach involved daily exposure of 128.7 ± 2.5 ppm average ozone concentration over 3 min on food spoilage organisms and whole food products like fruits and vegetables. The results showed that the inhibition approach can successfully inhibit the growth of both bacteria and mold species found in spoiled produce resulting in a greater than 5 log reduction of microbial colonies. Further research is needed to determine how much of the reduction in spoilage microbes is due to killing and how much is due to inhibition of growth. Energy required to power the reactor for 3 min daily was calculated to be 0.39 ± 0.06 kJ/day. Finally, ozone therapy through 3-min daily exposure on whole food products at room temperatures and under refrigerated conditions showed the potential of ozone therapy to at least double the shelf life of food products. Based on the experiments done during this study, we found 3-min periodic exposure to the APM is the most effective strategy for increasing shelf life. Future research plan includes a complete study of the effect of periodic ozone exposure on the sensory and nutritional values of produce products.

## Patents

This research outcome is part of a US patent 10,651,014 issued on May 12, 2020.
